# A congruent phylogenomic signal places eukaryotes within the Archaea

**DOI:** 10.1098/rspb.2012.1795

**Published:** 2012-10-24

**Authors:** Tom A. Williams, Peter G. Foster, Tom M. W. Nye, Cymon J. Cox, T. Martin Embley

**Affiliations:** 1 Institute for Cell and Molecular Biosciences, University of Newcastle, Newcastle upon Tyne NE2 4HH, UK; 2 Department of Life Sciences, Natural History Museum, London SW7 5BD, UK; 3 School of Mathematics and Statistics, University of Newcastle, Newcastle upon Tyne NE1 7RU, UK; 4 Centro de Ciências do Mar, Universidade do Algarve, Campus de Gambelas, 8005-139 Faro, Portugal

**Keywords:** phylogenetics, eukaryotes, evolution, tree of life

## Abstract

Determining the relationships among the major groups of cellular life is important for understanding the evolution of biological diversity, but is difficult given the enormous time spans involved. In the textbook ‘three domains’ tree based on informational genes, eukaryotes and Archaea share a common ancestor to the exclusion of Bacteria. However, some phylogenetic analyses of the same data have placed eukaryotes *within* the Archaea, as the nearest relatives of different archaeal lineages. We compared the support for these competing hypotheses using sophisticated phylogenetic methods and an improved sampling of archaeal biodiversity. We also employed both new and existing tests of phylogenetic congruence to explore the level of uncertainty and conflict in the data. Our analyses suggested that much of the observed incongruence is weakly supported or associated with poorly fitting evolutionary models. All of our phylogenetic analyses, whether on small subunit and large subunit ribosomal RNA or concatenated protein-coding genes, recovered a monophyletic group containing eukaryotes and the TACK archaeal superphylum comprising the Thaumarchaeota, Aigarchaeota, Crenarchaeota and Korarchaeota. Hence, while our results provide no support for the iconic three-domain tree of life, they are consistent with an extended eocyte hypothesis whereby vital components of the eukaryotic nuclear lineage originated from within the archaeal radiation.

## 1. Introduction

The early evolution of eukaryotes remains a fascinating and poorly understood period in the history of life. Eukaryotic cell structure is remote from that of Archaea and Bacteria, with features such as the nucleus, endomembrane system and associated organelles that have no obvious prokaryotic homologues [1]. As a result, hypotheses on eukaryotic origins have been motivated by comparisons of the small number of homologous gene sequences, particularly those of ribosomal RNA (rRNA) and protein-coding genes involved in nucleic acid replication, transcription and translation—the so-called ‘informational genes’ or ‘functional core of genomes’—that are conserved between eukaryotes, Archaea and Bacteria [2–9]. The rooted three-domains tree of life [2,9], in which the eukaryotic nuclear lineage is the sister group to a monophyletic Archaea comprising two major groups, the Euryarchaeota and Crenarchaeota, is probably the dominant paradigm for eukaryotic origins and it appears in many textbooks. However, other published phylogenies have suggested that eukaryotes emerged from within an already diversified archaeal radiation as the sister group to one of the several extant archaeal lineages [5,6,8,10,11]. The best known of these hypotheses is probably the eocyte hypothesis [5,6,10,12], which places eukaryotes as the sister group of the Crenarchaeota, a group also known as the eocytes.

Taxon sampling is one of the most important determinants of accurate phylogenetic estimation [13,14], and past attempts to resolve the origin of eukaryotes have been hindered by the relatively poor sampling of Archaea by genome sequencing. However, the discovery that uncultured Archaea play major roles in global nutrient cycles [15] has led to a number of sequence-based environmental surveys, which have improved sampling of Archaeal lineages. Recently discovered groups include the Thaumarchaeota [16], Aigarchaeota [17] and Korarchaeota [18]. Phylogenetic analyses suggest that all of these groups are more closely related to the Crenarchaeota than to the Euryarchaeota. Accordingly the name ‘TACK superphylum’ was recently proposed [19] to contain the Thaumarchaeota, Aigarchaeota, Crenarchaeota and Korarchaeota.

Although a consensus is emerging on the monophyly of the TACK superphylum [5,19,20], the relationships among its constituent lineages, and the relationship of the group as a whole to eukaryotes, remain unclear. Robust phylogenetic support for an origin for eukaryotes within, or as a sister group to, a characterized archaeal clade would be extremely exciting, because features shared between eukaryotes and extant archaeal members could inform a reconstruction of the ancestral eukaryote and provide insights into the early stages of eukaryotic evolution. The first author to draw a link between eukaryotes and any member of the TACK superphylum was Lake [7,10], whose eocyte hypothesis proposed a sister-group relationship between eukaryotes and the few Crenarchaeotes, or eocytes, which were known at that time; this relationship was also recovered by Cox *et al.* [6]. More recent phylogenetic analyses with extended taxonomic samplings have united eukaryotes with a clade comprising the Crenarchaeota plus Thaumarchaeota [5], with the Thaumarchaeota alone [21], or as part of an unresolved eukaryotes plus TACK supergroup [19]. All of these topologies are consistent with an eocyte hypothesis broadened in scope to include the newly discovered lineages. By contrast, a supertree analysis of single-copy protein families found in Bacteria, Archaea including Crenarchaeota, and eukaryotes, was interpreted to reject the eocyte hypothesis in favour of alternative hypotheses whereby eukaryotes emerge from within the Euryarchaeota [22,23]. Yet another analysis [24] has suggested that archaeal genes in eukaryotes derive from an ancient, probably extinct and in any case unknown, archaeal lineage.

As well as sparse taxon sampling, phylogenetic analyses attempting to infer ancient relationships face difficulties in the identification of reliable and informative phylogenetic markers. In addition to rRNA, empirical and simulation studies suggest that slow-evolving protein sequences conserved between Bacteria, Archaea and eukaryotes should also contain useful phylogenetic information [5]. However, individual proteins often resolve ancient divergences only weakly, leading to the practice of concatenating multiple alignments to increase statistical power. Since phylogenetic methods assume that these concatenated alignments evolve under a single topology, any horizontal gene transfers affecting the genes in the concatenation could lead to systematic phylogenetic error [25]. Horizontal transfer is now recognized as a frequent and important process in the evolution of all life forms [26], and failure to explicitly deal with its effects in inter-domain datasets could lead to a significant error in the inference of the relationship between Archaea and eukaryotes. At the same time, current phylogenetic methods will not necessarily recover correct or consistent relationships, even when all of the genes being analysed evolved on the same tree [27]. This is because lack of fit between the sequence data and the evolutionary model used can lead to systematic topological error. The use of appropriate evolutionary models is important in reducing the effects of phylogenetic artefacts such as long-branch attraction (LBA) [28,29], especially in the case of ancient relationships for which the phylogenetic signal may be weak.

Here, we have compared the support for current hypotheses of the relationship between Archaea and eukaryotes from rRNA and protein datasets representing the informational genes [2–9]. We included an expanded sampling of the emerging TACK superphylum of Archaea and used formal tests of topological congruence [30,31] to identify and characterize the distinct phylogenetic signals present in our alignments of conserved protein-coding genes. Posterior predictive simulations [32] were used to assess the fit of several different evolutionary models to our datasets, and the effect of model fit on inferred levels of phylogenetic incongruence was investigated by analysing sets of distances between trees. Our analyses consistently support the monophyly of eukaryotic informational genes with the TACK superphylum, but do not confidently identify the nearest neighbour of eukaryotes within this group. By contrast, we find no support for a euryarchaeal origin for eukaryotes or for the three-domains tree. With improved archaeal sampling, trees consistent with a broadly defined eocyte hypothesis are recovered both with standard and with more complex evolutionary models and for all subsets of data.

## 2. Results and discussion

### (a) The effect of new archaeal sequences on ribosomal RNA trees

Historically, rRNA has been the pre-eminent molecular marker for studies of ancient evolutionary events, and conflicting topologies inferred from rRNA genes have driven much of the debate on the deep structure of the universal tree [2,7]. In previous analyses [5,6,8,11,33], support from rRNA genes for the three-domains or eocyte hypotheses depended on the substitution model used: the simpler models generally gave a three-domains tree, whereas the more complex ones—for example, the node-discrete rate and composition heterogeneity (NDRH + NDCH) and CAT models [5]—gave an eocyte tree. These differences have been interpreted in terms of model fit, with the NDRH + NDCH and CAT models, for example, accounting for properties of the sequence alignment that are poorly anticipated by single-matrix models such as the general time reversible (GTR) model. In particular, site-specific selective constraints are not explicitly modelled by GTR, which assumes that the probability of change between any two nucleotides is the same at any site in the alignment. By contrast, comparisons of real sequence data strongly suggest that the phenotypic effect of a particular substitution, and therefore the evolutionary rate, depends on the function and biochemical context of the site [34]. Poor modelling of this substitution process makes GTR vulnerable to LBA, a well-characterized phylogenetic artefact in which parallel (convergent) substitutions along long branches of the phylogeny are misinterpreted as synapomorphies, causing these branches to group together [29,35]. A number of authors [6,8,11] have previously suggested that archaeal monophyly, and hence the three-domains tree, was the result of an attraction between the very long branches leading to the bacteria and eukaryotes. Interestingly, the CAT model, which models site-specific substitution rates with per-site frequency profiles and is reported to deal with LBA more efficiently [28], recovered a topology consistent with the eocyte hypothesis [5].

**Figure 1.**
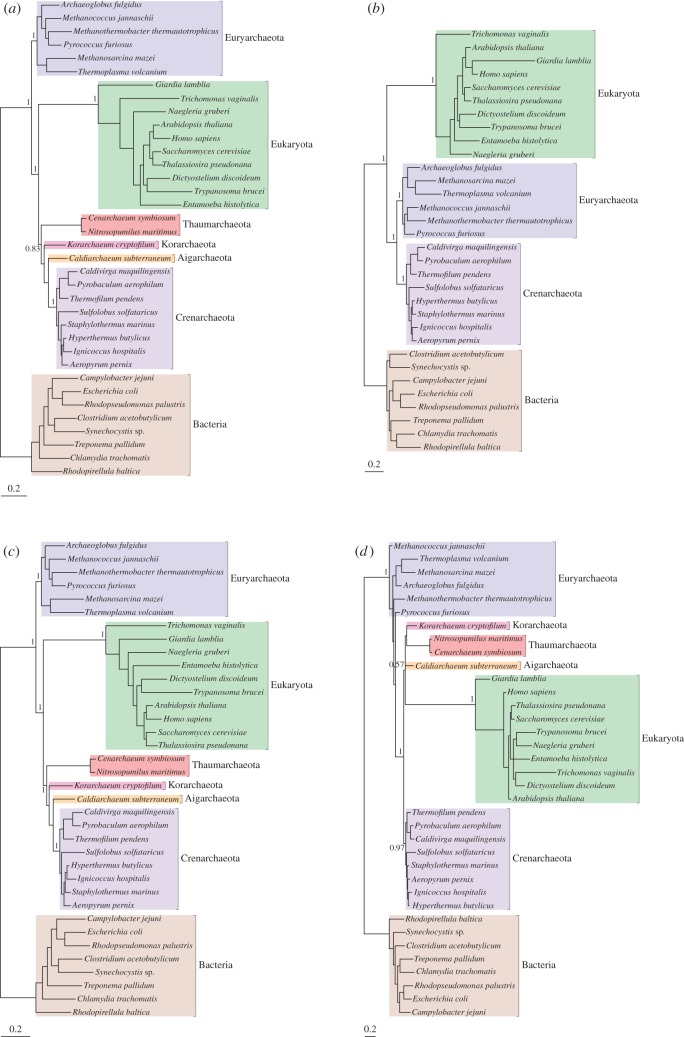
Phylogenies of Bacteria, Archaea and eukaryotes inferred from concatenated rRNA. (*a*) A Bayesian phylogeny of Bacteria, Archaea and eukaryotes inferred under the GTR model, showing an eocyte-like topology in which eukaryotes emerge from within the Archaea with maximal support (posterior probability (PP) = 1). (*b*) Removal of recently characterized archaeal groups (the Thaumarchaeota, Aigarchaeota and Korarchaeota) converts this tree into a canonical three-domains topology, again with maximal support (PP = 1), indicating that sampling plays an important role in the resolution of these ancient relationships. Analyses of the full dataset using the better-fitting NDRH + NDCH (*c*) and CAT (*d*) models recover maximally supported eocyte-like topologies; these models also recover eocyte-like topologies on the reduced dataset, without the TAK sequences (see the electronic supplementary material, figure S1). Branch lengths are proportional to substitutions per site.

To investigate the effect of new Thaumarchaeota, Aigarchaeota and Korarchaeota sequences on resolution of the deep branches of the tree of life, we built alignments of the large subunit (LSU) and small subunit (SSU) rRNA genes from 36 species of Bacteria, Archaea and eukaryotes. These alignments were based on those of Foster *et al.* [5] but were updated to reflect the recent improvement in sampling of free-living microbial eukaryotes (*Naegleria gruberi*) and TACK superphylum members (*Korarchaeum cryptofilum* and *Caldiarchaeum subterraneum*). The phylogenetic signal in the LSU and SSU alignments was determined to be congruent by two complementary methods [30,31], enabling us to concatenate them for further analysis. We used RAxML v. 7.2.8 [36] to build a maximum likelihood bootstrap tree for the combined LSU + SSU alignment, optimizing a GTR model separately on each partition as indicated by jModelTest [37]. We also built Bayesian phylogenetic trees under the GTR and CAT models in PhyloBayes, and the NDRH + NDCH model in p4. In the following discussions of the relationships between eukaryotes and Archaea, we have, like others [2,9], taken the root of the universal tree to be either within, or on the branch leading to, the Bacteria [38–40]. This position remains tentative (see [6] for discussion), but the three-domains and eocyte-like trees are actually incompatible wherever the root lies. As in previous work [5], our analyses using the better-fitting NDRH + NDCH and CAT models (figure 1*c,d*) recovered an eocyte topology. However, in contrast to previous results [5], our analyses also recovered a strongly supported eocyte topology with the GTR model (figure 1*a*).

Our failure to obtain a three-domains tree, even with the data-homogeneous (non-mixture) GTR model, was surprising given previous results, so we performed several phylogenetic experiments to investigate the cause. First, we used posterior predictive simulations [32] to evaluate the fit of the GTR, NDRH + NDCH and CAT models to the rRNA dataset (see the electronic supplementary material, table S1). These tests indicated that the GTR model is a poor fit to the dataset with respect to base composition and site-specific biochemical diversity. The more complex models were each able to account for some, but not all, of the features of the rRNA alignment. Thus, the CAT model was much better than GTR at modelling the site-specific features of the substitution process, but it failed to account for the compositional heterogeneity present in the data. Fit with respect to composition was achieved with the NDRH + NDCH model, which allows composition to vary over the tree [41]. These results are similar to those reported previously, where the NDRH + NDCH and CAT models outperformed the single-matrix GTR model for model fit [5], and they suggest that the newfound support from GTR for an eocyte-like topology is not the result of improved model fit with the updated rRNA alignment.

Since we had used a conservative masking protocol (Gblocks with the default parameters) in constructing our original alignment, we investigated whether properties of the alignment had influenced the result. We used an alternative alignment masking protocol (the ‘automated1’ option in TrimAl [42]) that retained substantially more sites (2227 versus 1184 positions), and reanalysed our data using the same methods as previously. All three models recovered an eocyte topology from this alignment (see the electronic supplementary material, figure S1). Removal of the Thaumarchaeota, Aigarchaeota and Korarchaeota sequences, however, produced a three-domains tree under the GTR model (figure 1*b*), although an eocyte topology was still recovered under the better fitting NDRH + NDCH and CAT models (electronic supplementary material, figure S1). These results suggest that increased sampling of divergent members of the TACK group improved resolution of the inner nodes of the tree of life, leading to the recovery of an eocyte tree even with the simpler model of nucleotide substitution.

Although trees inferred using all three models produced eocyte topologies (i.e. in which the TACK sequences clustered with eukaryotes to the exclusion of the Euryarchaeota), they also displayed significant topological differences among major archaeal groups (figure 1). For example, in the GTR and NDRH + NDCH trees, the Euryarchaeota are monophyletic with maximum support, whereas in the CAT tree they were paraphyletic, also with maximum support. To increase the number of characters brought to bear on these questions we turned our attention to conserved protein-coding genes.

### (b) Support from conserved protein-coding genes for hypotheses of eukaryotic origins

**Figure 2.**
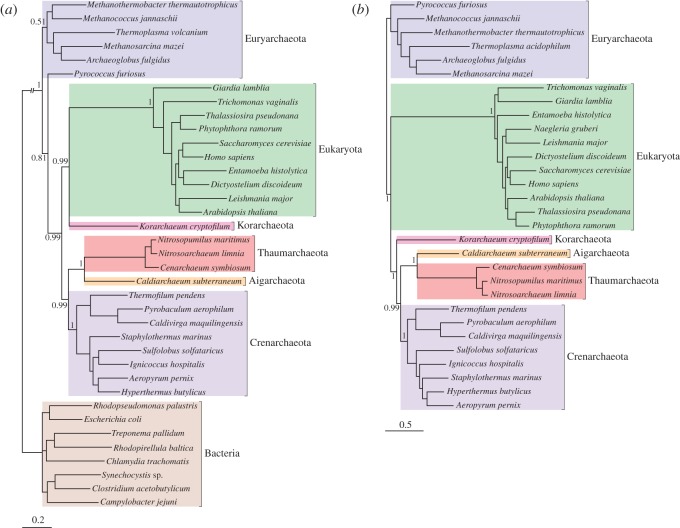
Phylogenies of Bacteria, Archaea and eukaryotes inferred from conserved protein-coding genes. (*a*) A phylogeny inferred from 29 concatenated proteins conserved between Bacteria, Archaea and eukaryotes. An eocyte topology was recovered with strong (PP = 0.99) support. In this phylogeny, the eukaryotes emerge as the sister group of *Korarchaeum*, nested with the TACK superphylum. (*b*) A phylogeny inferred from 63 concatenated proteins shared between Archaea and eukaryotes. The position of the root is not explicitly indicated. However, based on the result from (*a*) and the electronic supplementary material, table S4, it is likely to be either within, or on the branch leading to, the Euryarchaea. If this position is correct, then the tree shows the eukaryotes emerging as the sister group to the TACK superphylum, including *Korarchaeum*. These trees were inferred using the CAT model in PhyloBayes. Branch lengths are proportional to substitutions per site, except the truncated bacterial branch in (*a*).

We assembled two protein datasets: a set of 29 proteins conserved across Bacteria, Archaea and eukaryotes (29BAE), in order to compare support for the three-domains versus the eocyte hypotheses for eukaryotic genes; and a larger set of 64 genes conserved in our sample of Archaea and eukaryotes (64AE) for investigating the in-group relationships between the eukaryotes and specific archaeal lineages. These conserved genes (see the electronic supplementary material, tables S2 and S3) are mainly involved in information processing (DNA replication, transcription and translation), and includes those that have been called the ‘genealogy-defining core’ of cellular life forms [43] or the ‘functional core of genomes’ [9]. It has been suggested that these genes may be more resistant to horizontal gene transfer (HGT) than the rest of the genome because their gene products have complex cellular interactions [44,45]. Nonetheless, information-processing genes are not immune to HGT [46], and as the number of markers that are concatenated to build a phylogeny increases, so too does the probability that at least some of them will be affected by HGT. Since phylogenetic methods assume a single underlying topology, concatenation of genes with different evolutionary histories could potentially result in serious systematic error [47]. To account for these difficulties, we used two complementary methods to test the congruence of these information-processing genes: Concaterpillar [31] and Conclustador [30]. Interestingly, the two methods disagreed on the level of incongruence in our protein datasets: Conclustador, which uses spectral clustering of Euclidean distances to define sets of topologically similar trees, inferred a single congruent set from each of the 29BAE and 64AE datasets, whereas Concaterpillar, which implements a hierarchical likelihood ratio test, inferred a number of congruent subsets (five in 29BAE and 15 in 64AE) in each case. To characterize the range of phylogenetic signals identified in our protein datasets, we built trees for each set of genes inferred to be congruent by either of the methods. In the case of the 64AE dataset, we also obtained strong evidence from a third approach (see below) that one of the genes was incongruent; this gene was removed from the complete concatenation, resulting in a 63AE dataset. Each congruent set was analysed as a single partition using the CAT family of phylogenetic models [34,48]. We used the full CAT model for concatenates that were over 1000 amino acids in length, and the CAT20 model for those that were shorter. CAT20 is a variant of the more flexible CAT model that contains an empirical profile mixture of 20 components inferred from the homology-derived structures of proteins alignment database, analogous to the empirical substitution matrices in standard models such as LG. It is optimized for use on smaller alignments, where CAT may perform poorly [48]. Phylogenies inferred from the complete datasets are presented in figure 2; phylogenies inferred from the Concaterpillar-derived congruent subsets are provided in the electronic supplementary material, figures S2 and S3. The support from all these analyses for current hypotheses on eukaryotic origins is summarized in the electronic supplementary material, tables S4 and S5.

Our analyses including bacterial outgroups consistently supported the monophyly of eukaryotes with the TACK superphylum of Archaea, to the exclusion of the euryarchaeotes, although the strength of support for this eocyte-like hypothesis varied with the subset of the data analysed (see figure 2 and the electronic supplementary material, tables S4 and S5). By contrast, we found no support for the three-domains hypothesis and the monophyly of Archaea from any of these analyses. While the monophyly of eukaryotes and the TACK superphylum was consistently recovered, the specific relationships within this clade were more ambiguous. The phylogeny inferred from the 63AE dataset recovered eukaryotes and the TACK superphylum as separate clusters (figure 2*b*); in contrast, the 29BAE dataset and the two largest Concaterpillar-derived congruent subsets inferred from the 64AE dataset supported the nesting of the eukaryotes *within* the TACK superphylum, either as the neighbour of *Korarchaeum* or with the relationship unresolved (see figure 2*a* and the electronic supplementary material, tables S4 and S5). Given the sparse sampling of Korarchaeota and their relatives, and the long branch leading to eukaryotes, this finding must be treated with caution [14]. In particular, when the bacterial sequences were removed from the 29BAE dataset and the analyses were repeated, the relationship between eukaryotes, *Korarchaeum* and the rest of the TACK superphylum collapsed to a trichotomy, suggesting that the *Korarchaeum*/eukaryote link is not strongly supported (see the electronic supplementary material, figure S6). Further Korarchaeal genome sequences are likely to be very informative about this part of the tree of life. It is interesting to note that we consistently recovered a strongly supported Thaumarchaeota/Aigarchaeota clade within the TACK group, confirming the relationship between these groups [20] and suggesting that they do not represent the earliest-diverging archaeal lineage [16,49]; in our trees, the eukaryotes and the TACK superphylum consistently form a monophyletic group to the exclusion of euryarchaeotes (figure 2, electronic supplementary material, figure S2).

With the exception of *Korarchaeum*, our analyses did not provide support for a specific relationship between any members of the TACK superphylum and eukaryotes. In particular, we found no strong support for a specific relationship between the Thaumarchaeota and the eukaryotes, as has recently been suggested [21] (see the electronic supplementary material, table S5). Further, our results were not compatible with a sister-group relationship [22] between the eukaryotes and the Thermoplasmatales, a group of euryarchaeotes. In our trees, *Thermoplasma* consistently grouped within the euryarchaeotes, with no significant support from any analysis for a *Thermoplasma*/eukaryote clade (see the electronic supplementary material, table S5). To determine the reason for this disagreement, we compared our 64-gene dataset with that originally used to suggest the *Thermoplasma* link [22]. Of the 5741 protein families examined in that study, 41 contained both a member of the Thermoplasmatales and at least one eukaryote; the support for a *Thermoplasma*/eukaryote link comes from 12 of these families in which the eukaryotes and Thermoplasmatales form a clade. Only one of these 12 protein families (Cbf5, encoding an rRNA pseudouridine synthase) was included in our 64-gene dataset; the others were not included in our analyses because of their patchy distribution across eukaryotes and Archaea. In the case of Cbf5, our single-gene phylogeny did not recover a *Thermoplasma*/eukaryote relationship (see the electronic supplementary material, figure S5), and it was only weakly supported (21% maximum likelihood bootstrap value) in the published tree [22]. In the eight cases where more than a single eukaryotic sequence was included in a protein family, we built new phylogenetic trees, adding in orthologous sequences from the TACK genomes that have been sequenced since 2007 (see the electronic supplementary material, table S6). We recovered a weakly supported *Thermoplasma*/eukaryote relationship in three trees: those based on a tRNA pseudouridine synthase (posterior probability (PP) = 0.79), a wbutosine synthesis protein (PP = 0.72) and an RNA-binding protein (PP = 0.53); see the electronic supplementary material, figure S5. In the tree built from the wbutosine synthesis protein, the Thermoplasmatales clustered outside of the euryarchaeal radiation (PP = 0.99), with their closest neighbours being the Crenarchaeote *Thermofilum pendens* and the Aigarchaeote *Caldiarchaeum subterraneum*. Since the Thermoplasmatales are generally recovered within the Euryarchaea (see figure 2 of this paper, or [20]), their position in this tree is unusual, making it unlikely that we can draw strong inferences from these data. In summary, our analyses of concatenated proteins and re-analyses of single-gene trees found no compelling support for a specific role for *Thermoplasma* in eukaryotic origins.

### (c) The effect of evolutionary model on inferred levels of phylogenetic incongruence

**Figure 3.**
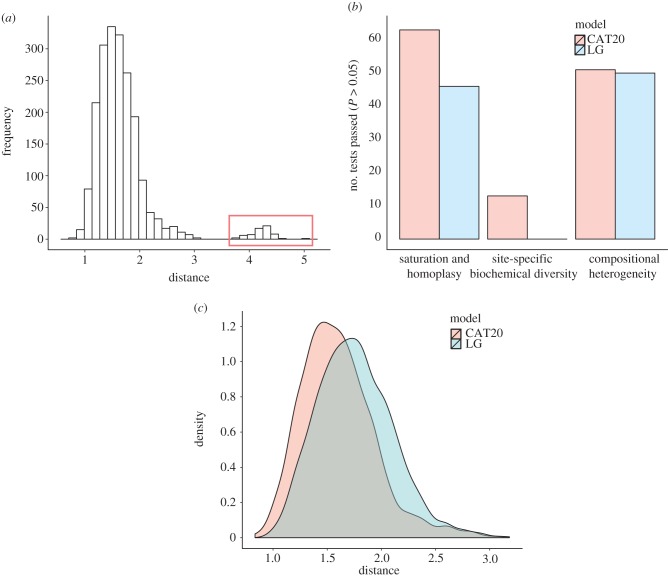
Analysing incongruence using a novel measure of distance between gene trees. We used distributions of pairwise geodesic distances between gene trees to compare levels of incongruence inferred under different evolutionary models. (*a*) The distribution of distances under a single model (CAT20) can be used to identify obvious outliers corresponding to highly incongruent gene trees; a single gene was responsible for the peak highlighted in red, and was removed from subsequent analyses. (*b*) Overview of model-fitting tests (posterior predictive simulations) for each gene in the 64AE dataset. The height of the bars indicates the proportion of genes that ‘passed’ a test under a particular model; we said that a test was passed when the value of the test statistic on the real data fell within the central 95% of the distribution of values produced by posterior predictive simulation. The results suggest that CAT20 fits better than LG, successfully accounting for the observed levels of saturation and homoplasy in all but one of the alignments. Both models do a poor job of modelling the site-specific selective constraints in our dataset, although again CAT20 performs better than LG (13 passes as opposed to 0). (*c*) Comparison of the distance distributions inferred under the CAT20 and LG models. The trees inferred under the better-fitting CAT20 model are significantly more congruent than those inferred under LG (mean distance: 2.68 versus 3.22, *p* < 0.0001). The significance of this difference was assessed using a permutation test that took the correlations between pairwise distances into account (see §4). These results suggest that a significant portion of the incongruence in this dataset of informational genes can be attributed to model misspecification, rather than genuinely distinct evolutionary histories.

The distinct phylogenetic signals we identified in our protein datasets could have resulted from genuinely different gene histories (HGT) or from phylogenetic error. Current evolutionary models make assumptions about the data, such as homogeneity of the substitution process across sites, or of composition across the tree, that are often violated, potentially leading to topological error. The variable position of *Korarchaeum* in our analyses may reflect these issues. When analysing different datasets (see figure 2 and the electronic supplementary material, table S5) or using different phylogenetic models (see the electronic supplementary material, figure S6), *Korarchaeum* was either recovered as the closest archaeal relative of eukaryotes or as an early-diverging member of the TACK superphylum. Furthermore, there were no apparent patterns in the functions or identities of protein complexes represented by the different congruent subsets of genes supporting one placement or another (see the electronic supplementary material, tables S7 and S8). For example, individual components of the large and small ribosomal subunits were found in different congruent sets. These results suggested that at least some of the incongruence in our protein datasets was because of phylogenetic artefacts. If this is the case, then the choice of evolutionary model should affect inferred levels of incongruence, because current models vary in their sensitivity to systematic phylogenetic error [28]. To evaluate this possibility, we developed a method for comparing levels of incongruence under different evolutionary models that uses distributions of geodesic distances [50] between trees (see figure 3 and the electronic supplementary material). These distances provide a continuous measure in tree space that incorporates differences in both branch lengths and tree topologies. We inferred gene trees for each gene in the 64AE dataset using LG, which was the best-fitting single-matrix model in each case, and CAT20 which, as discussed above, is an empirical variant of the CAT model which is more suitable for short single-gene alignments. For each model, we calculated all pairwise geodesic distances between trees. Although we calculated these distances in order to compare different models, the distance distributions under each model already contain some useful information about congruence. For these 64 genes, the distributions had a marked hump in the tail (figure 3*a*) corresponding to a single, clearly incongruent gene tree (see the electronic supplementary material, figure S7); we removed this tree from subsequent analyses, resulting in the 63AE dataset. Interestingly, the trees inferred from the 64- and 63-gene concatenations were topologically identical, suggesting that—at least in this case—small amounts of incongruent data are overpowered by the dominant signal in large concatenations. Comparisons of model fit using posterior predictive simulations indicated that CAT20 was a better-fitting model than LG for the individual genes comprising the 63AE dataset (figure 3*b*), as has previously been observed on large samples of saturated amino acid alignments [48]. The mean squared geodesic distance between trees inferred under CAT20 was significantly lower than that inferred under LG (2.68 versus 3.22; *p* < 0.0001; figure 3*c*), suggesting that trees inferred under the better-fitting model were more congruent. This result suggests that a significant portion of the incongruence in the dataset can be attributed to model misspecification, as opposed to genuinely discordant evolutionary histories. It will be interesting to evaluate whether this result also applies to larger-scale, less strictly filtered datasets. In the present case, disagreement among the larger congruent subsets was associated with the placement of *Korarchaeum*, with broad support for an eocyte-like, rather than a three-domains tree from the majority of genes and subsets (see the electronic supplementary material, tables S4 and S5).

## 3. Conclusions

Under the three-domains hypothesis, important components of the eukaryotic genetic machinery were vertically inherited from a common ancestor shared with Archaea, and this relationship is taken to explain the shared properties of both groups. In an eocyte-like scenario, those same eukaryotic components were vertically inherited from an ancestor that was already an archaeon, and the phylogenetic position of this ancestor could be particularly informative about the genetic and metabolic context of early eukaryotic evolution and for theories of eukaryotic origins [1]. Here, we have compared support for these hypotheses and others, using conserved components of the genetic machinery. With an updated sampling of archaeal diversity, we found no support for the three-domains hypothesis either from rRNA or protein-coding genes under any phylogenetic model. Instead, we detected a congruent phylogenetic signal that placed essential informational genes of the eukaryotic nuclear lineage within the archaeal radiation, sharing common ancestry with the TACK superphylum. The monophyly of eukaryotic genes with the TACK superphylum was consistently recovered but the specific relationships within this clade were not decisively resolved; in particular, we did not recover a sister-group relationship between the Thaumarchaeota and the eukaryotes, as recently proposed [21]. As such, we cannot discriminate between an origin for eukaryotic genes from within the TACK superphylum [19], or from a sister-group lineage. In contrast to a recent supertree study [22], we did not find any support for a role for *Thermoplasma* in eukaryotic origins. Intriguingly, members of the TACK superphylum encode homologues of genes that were previously thought to be eukaryote-specific, such as actin [51], the Cdv cell division machinery [52] and a ubiquitin protein modification system [17], although no single characterized TACK genome possesses all of these features. Although these genes have a patchy distribution in extant TACK genomes, it has been suggested that they could potentially have co-occurred in the ancestor of the clade [19], a scenario supported by evidence for extensive reductive evolution in the Archaea [53]. The recent report of a eukaryote-type tubulin in *Nitrosoarchaeum* [54] is particularly exciting because it implies that both actin [55] and tubulin might have already been present in an archaeal ancestor of eukaryotes. Thus, not only the core genetic machinery, but also core components of the eukaryotic cytoskeleton could have been inherited from a relative of the TACK Archaea.

## 4. Material and methods

### (a) Sequences and alignments

The rRNA and 29BAE protein alignments were based on those of Foster *et al.* [5], but updated with the relevant sequences from *Naegleria gruberi*, *Korarchaeum cryptophilum*, *Caldiarchaeum subterraneum* and *Nitrosoarchaeum limnia*. Sequences were aligned with Meta-Coffee, and poorly aligning regions were identified are removed using Gblocks or TrimAl, as described in the main text. To prepare the 64AE alignments, we performed clustering of the proteomes with the Markov Cluster algorithm of the selected taxa, and built maximum likelihood trees from the initial clusters to identify single-copy orthologues which were used for phylogenetic analysis. Further details of the sequence selection and alignment protocol are provided in the electronic supplementary material.

### (b) Congruence tests

We used Concaterpillar v. 1.5 [31] and Conclustador v. 0.1a [30] to test whether our single-gene alignments were congruent before concatenating them for phylogenetic analysis. In cases where these two methods disagreed, we built phylogenies for all of the congruent sets inferred by both methods and compared the results. To complement and expand upon these approaches, we developed a novel method for analysing the level of incongruence in a set of genes and for comparing incongruence between sets of trees inferred under different models using geodesic distances; this method is described in detail in the electronic supplementary material.

### (c) Phylogenetics

Best-fitting substitution models were chosen for the rRNA alignments using jModelTest [37]. For the protein alignments, single-matrix substitution models were chosen using the ProteinModelSelection script available from the RAxML website (http://www.exelixis-lab.org/). Maximum likelihood calculations were performed with RAxML v. 7.2.8 [36]. Bayesian Markov Chain Monte Carlo analyses were performed using the p4 (http://code.google.com/p/p4-phylogenetics/; Foster [41]) and PhyloBayes v. 3.3 [56] packages, which implement the range of more complex models used in our analyses. Convergence was assessed by comparing the results from independent runs, and model fit in the Bayesian analyses was evaluated using posterior predictive simulations [32]. Further details of the models and simulations used are provided in the electronic supplementary material.
